# A crystal plasticity FEM study of through-thickness deformation and texture in a {112} <111> aluminium single crystal during accumulative roll-bonding

**DOI:** 10.1038/s41598-019-39039-y

**Published:** 2019-03-04

**Authors:** Hui Wang, Cheng Lu, Kiet Tieu, Guanyu Deng, Peitang Wei, Yu Liu

**Affiliations:** 1 School of Mechanical, Materials and Mechatronic Engineering, University of Wollongong, New South Wales, 2522 Australia; 2 State Key Laboratory of Mechanical Transmission, Chongqing University, Chongqing, 400044 China

## Abstract

In this study, a crystal plasticity finite element method (CPFEM) model was used to study the deformation behaviour in an aluminium single crystal (1 1 2)[1 1 -1] processed by accumulative roll-bonding (ARB) up to 9 cycles. The simulation followed the real ARB process based on the developed finite element model. The predicted through-thickness texture matches well with the experimental observations. The deformation behaviours, in terms of crystal rotation, shear strain and slip system activation, in the first and second cycles (conventional rolling) were unidirectional, but the deformation was altered after ARB was applied from the third cycle onwards. Such alteration was found to be caused by the thickness position change and deformation discontinuity at interfaces, which were investigated in detail. The role that interfaces play became dominant over thickness position change as increasing ARB cycles.

## Introduction

Accumulative roll-bonding (ARB), a severe plastic deformation (SPD) method, has been extensively applied to fabricate ultra-fine or even nanocrystalline grained materials^1,2^. A large strain can be introduced into materials by repeating the standard 4-step procedure (wire-brushing, stacking, roll-bonding, and cutting)^3^. Due to large surface friction, a heterogeneous through-thickness deformation (e.g., texture, microstructure and strain) after each cycle has been experimentally revealed in ARB processed metals and alloys^1,2,4^, and the deformation behaviour is altered after the thickness position being changed between cycles (because of cutting-stacking). The through-thickness inhomogeneity in a cycle and deformation alteration between cycles make the deformation behaviour in ARB complicated.

Texture modelling has become a powerful tool to study plastic deformation and texture evolution, and various crystal plasticity (CP) models have been developed. The Taylor model^5^, ALAMEL model^4,6^ and viscoplastic self-consistent (VPSC) model^7–12^ have been used to simulate texture evolution in ARB processed polycrystals. Only textures at the centre and surface were predicted by the ‘uniform-field’ Taylor model in ref.^5^, while textures at the centre, surface and quarter were modelled in refs^4,6^. Textures of two-phase composites were studied by the so-called ‘mean-field’ VPSC model^8–12^. Intrinsic homogenization at different levels is assumed in these CP models, and accordingly, the predicted ARB textures in these studies^4–12^ are more in a sense of statistical manner. Through-thickness texture and its transition between cycles have not been explicitly revealed in simulations.

The crystal plasticity finite element method (CPFEM) model, a more sophisticated CP model, is more applicable to ARB, as suggested by Knezevic *et al*.^8^. The main advantage of the ‘full-field’ CPFEM model over those above mentioned CP models is no homogenization assumption^7^, in which the crystal plasticity constitutive model is incorporated into the finite element method (FEM) framework. The plastic deformation and texture evolution are fully coupled in the CPFEM model. The equilibrium and compatibility conditions between elements and between grains are reached by basic principles of mechanics^13^, so the CPFEM model can access both intra- and inter-grain deformation^14^. Being able to access the former (intra-grain deformation) means the CPFEM model can simulate the deformation texture in (strongly anisotropic) single crystals that is the material used in this study. Non-uniform through-thickness deformation and cutting-stacking in ARB would result in misorientation angles at the bonded interfaces^15,16^, as shown later in this study, and the deformation behaviour at the interfacial boundaries can be simulated by the CPFEM model because of its capability to access inter-grain interaction^8^.

The CPFEM model has been applied to study the plasticity at interfaces of two-phase composites fabricated by ARB^17–19^, but in these studies ARB was approximated by plain strain compression. Modelling the real ARB process is challenging^4^, since it is a discontinuous process. The ARB FEM model in ref.^20^ resulted in a large distortion of FEM mesh, which would cause convergence problems and restricted the ARB simulation to a low cycle number. A modelling method, mapping solution, has been used between ARB cycles to transfer the deformation solution from the deformed mesh to a new mesh^2,7^. However, the ARB simulation in ref.^2^ was based on an elasto-plastic material constitution law without considering texture, while a VPSC model was used in ref.^7^ but it only simulated up to 2 cycles. Li *et al*.^4^ modelled the texture at the surface, centre and quarter up to 5 cycles with the ALAMEL model, but the simulation did not follow the real multi-cycle ARB. In their paper, the deformation history of the first cycle based on the elasto-plastic constitution law was used to model texture in all 5 cycles, i.e., decoupled plastic deformation and texture evolution. According to the authors’ best knowledge, no CPFEM simulation following the real ARB has been conducted up to a large cycle number (≥5).

In the present study, a CPFEM model was adopted to predict plastic deformation and texture evolution in an ARB processed aluminium single crystal (1 1 2)[1 1 $\bar{1}$]. The simulation following the real ARB process was conducted up to 9 cycles, and the predicted through-thickness texture has been validated by the corresponding experimental observations. The deformation behaviour, in terms of strain, slip system activation, crystal rotation, and crystal orientation stability, in ARB was investigated.

## Modelling Details

**Figure 1:**
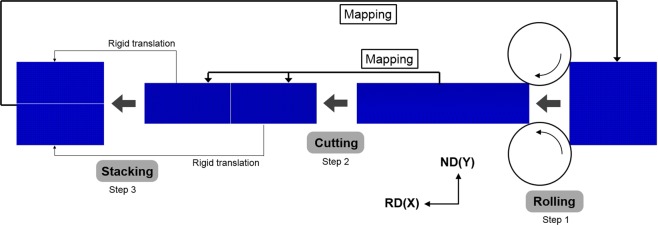
A schematic of ARB FEM model including ‘Rolling’, ‘Cutting’ and ‘Stacking’ three steps, and mapping solution was used to transfer the deformation solution from the deformed mesh to a new mesh.

Figure 1 shows the ARB FEM model, which is two dimensional under the assumption of plain strain condition. Similar to refs^2,7^, mapping solution, a built-in remeshing technique of the commercial FEM code ABAQUS, was used between cycles. The deformed mesh was replaced by a new mesh after mapping solution, while the deformation solution (e.g., stress, strength and crystal orientation) in the new mesh was interpolated from the distorted mesh. During the interpolation, the solution at nodes of the deformed mesh was firstly obtained by extrapolating the values of integration points and then the solution at nodes was averaged over all elements around the nodes in the new mesh. The discrepancy caused by the interpolation has been checked in this study by comparing the solution before and after mapping solution, and it was reduced to an acceptable level by refining the FEM mesh.

In ARB experiments, the wire brushing would result in a superficial layer of finely grained structures on the sheet surfaces^21,22^, and this layer plays an important role in plastic deformation at a small scale^23^, but its influence to the global texture is limited. The ‘Wire-brushing’ step was thus omitted in this ARB FEM model. The two stacked sheets in ARB experiments are usually jointed by metal wire or welding before roll-bonding, and accordingly, the relative displacement between the two stacked sheets is prohibited or very small. Moreover, the wire-brushing would cause a high surface roughness of the sheets to be stacked, and the high roughness would greatly reduce the relative slide between the two stacked sheets when the compression stress along the normal direction (ND) increases in the rolling bite. Therefore, rolling was used to replace roll-bonding in this ARB FEM model. The single-layered sheet was rolled at a 50% reduction in the first step ‘Rolling’ (Fig. 1), and then it was mapped (mapping solution) into two separate ones in the second step ‘Cutting’. The two separate sheets were stacked by rigid translation along the rolling direction (RD) and ND in the third step ‘Stacking’, so no deformation was introduced during this translation, and then the stacked sheets were mapped into another single-layered one in preparation for the next cycle. The boundary conditions, which can be simply described as the sheet deformed by rotating rolls via surface friction, were reapplied after mapping solution at the beginning of the next cycle.

The simulation was specifically designed to match the ARB experiment conducted by Kashihara *et al*.^16^. The rolls were considered as analytical rigid bodies with a diameter of 310 mm. After testing various friction coefficients, 0.12 was chosen as the coefficient of friction between the sheet and rolls, because it provided the best match of textures, and it is also the suggested value for lubricated ARB^24^. It was conventional rolling in the first and second cycles, the same as the experimental procedure^16^, where the starting sheet (4 mm in thickness) was rolled to 2 mm by a 50% reduction in the first cycle and then to 1 mm in the second cycle. The deformed sheet after the first cycle was directly mapped to a new FEM mesh at the beginning of the second cycle, without ‘Cutting’ and ‘Stacking’ steps. ARB was applied from the third cycle onwards, and the two stacked sheets made the starting thickness be 2 mm from 3- to 9-cycle. The element type was CPE4R, which can provide efficient and fast numerical formulation. Enhanced hourglass control was used to increase the resistance to the hourglassing problem and provide more accurate displacement solution. The shape of elements was square, i.e., equal length along the RD and ND. After examination of mesh division, the whole thickness was divided into 40 elements in the first four cycles, and then the element number was doubled after every two cycles. The initial orientation (1 1 2)[1 1 $\bar{1}$] was assigned to all elements (or integration points) at the very beginning of the simulation, since the material used in the experiment^16^ was a single crystal (1 1 2)[1 1 $\bar{1}$]. No assignment of crystal orientations was conducted in the following cycles, in which the starting texture (of 2-cycle onwards) was obtained (from the last cycle) by mapping solution. The orientations can rotate in different paths though they have the same initial position (1 1 2)[1 1 $\bar{1}$]. The whole through-thickness texture was modelled in this study.

**Figure 2:**
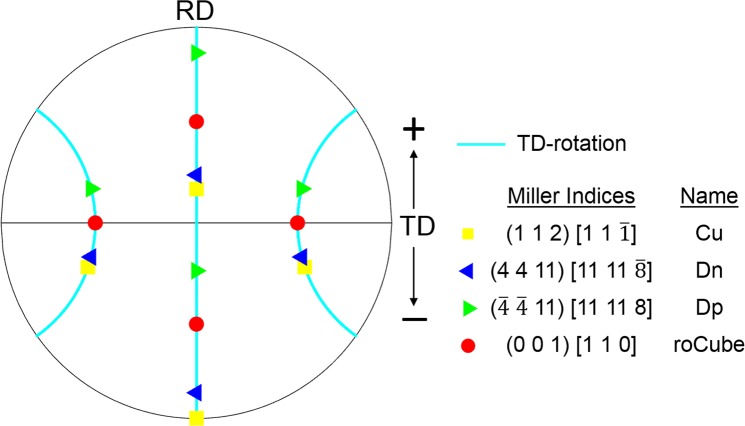
A (1 1 1) pole figure shows the relation between four crystal orientations (Cu, Dn, Dp and roCube) and TD-rotation, and positive and negative TD-rotation are indicated by ‘+’ and ‘−’, respectively.

The crystal rotation angle, i.e., misorientation between final orientation and initial orientation (1 1 2)[1 1 $\bar{1}$], in each element was calculated following the Bunge’s convention, which was further partitioned into rotation about the RD, transverse direction (TD), and ND according to the method proposed in ref.^25^. TD-rotation is strongly dominant over RD- and ND-rotation, and thus only TD-rotation among these three components will be presented and discussed in the following text. Figure 2 shows the positions of four orientations used in this study, between which a TD-rotation is the difference, as shown by the rotation path of TD-rotation. The initial orientation (1 1 2)[1 1 $\bar{1}$] (Cu) would change to (4 4 11)[11 11 $\bar{8}$] (Dn) by rotating 8° about the TD in the positive direction, and to (0 0 1)[1 1 0] (roCube) and ($\bar{4}$ $\bar{4}$ 11)[11 11 8] (Dp) by further increasing TD-rotation to 37° and 62°, respectively.

The kinematical scheme and hardening model used in the CPFEM model are given in Appendix A and B, respectively, and they were implicitly incorporated into ABAQUS/Standard ver.6.9 by the user-defined material subroutine. The slip plane is {1 1 1} and slip direction is <1 1 0> in aluminium of FCC structure, and their combinations generate 12 potentially activated slip systems. This CPFEM model has already been successfully applied to other SPD techniques such as equal channel angular press (ECAP)^26^ and high pressure torsion^27^, and also rolling^28^, and satisfactory textures have been obtained.

## Results and Validation

The thickness location is defined as *t*/*t*0, where *t* is the distance from the upper surface to a thickness position and *t*0 is the whole thickness. Therefore, *t/t0* = 0 corresponds to the upper surface and 1 to the lower surface.

**Figure 3:**
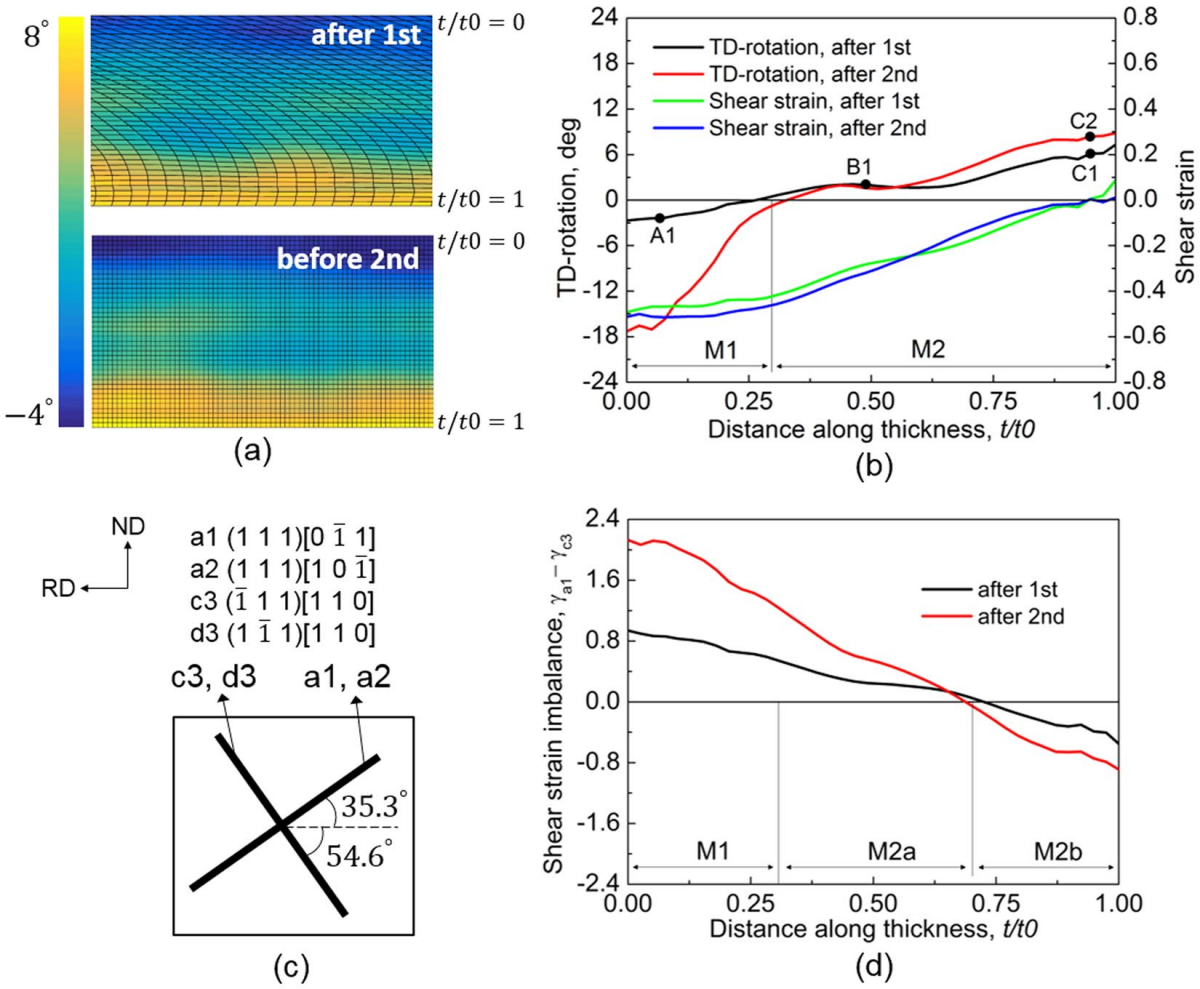
(**a**) Distribution of FEM mesh and cumulative TD-rotation after 1-cycle and before 2-cycle. (**b**) Through-thickness shear strain and cumulative TD-rotation after 1- and 2-cycle, where the shear strain is that evolved in a single cycle, but not cumulated with ARB cycles. (**c**) Distribution of four primarily activated slip systems on the RD-ND plane before rolling. (**d**) Distribution of imbalance between cumulative *γ*_*a*1_ and *γ*_*c*3_ (=*γ*_*a*1−_*γ*_*c*3_) after 1- and 2-cycle.

It is conventional rolling in the first and second cycles. Figure 3a shows the distribution of FEM meshes and cumulative TD-rotation after 1-cycle and before 2-cycle. It is clear that the distortion of FEM mesh in 1-cycle was eliminated by mapping solution before 2-cycle, but the deformation solution, e.g., TD-rotation in Fig. 3a, was preserved. The TD-rotation is almost uniform along the RD, but it varies obviously through the thickness. Figure 3b shows the through-thickness cumulative TD-rotation after 1- and 2-cycle, where the TD-rotation angle is the average value of all elements (along the RD in steady state deformation region) at the same thickness position. The TD-rotation divides the whole thickness into two matrix bands, M1 (*t*/*t*0 = 0 to 0.25) and M2 (*t*/*t*0 =  0.25 to 1), with negative and positive rotation, respectively. The TD-rotation angle after 1-cycle is very low, and after 2-cycle it continued to increase greatly in the negative direction in M1, while a small increase in the positive direction developed in M2. The division of M1 and M2 in 1- and 2-cycle is slightly different, which was probably caused by the change of rolling bite geometry^29^. Shear strain *γ*_*XY*_ evolved in a cycle alone, not cumulated with ARB cycles, is also shown in Fig. 3b. The shear strain *γ*_*XY*_ is very high at the upper surface in 1- and 2-cycle, while it is almost zero at the lower surface. The asymmetrical distribution of shear strain indicates a large slide occurred between the sheet and rolls under lubricated rolling conditions.

In this study, the activated slip systems are a1 (1 1 1)[0 $\bar{1}$ 1], a2 (1 1 1)[1 0 $\bar{1}$], c3 ($\bar{1}$ 1 1)[1 1 0], and d3 (1 $\bar{1}$ 1)[1 1 0], which is consistent with the experimental study^16^. The slip system a1 and a2 are co-planner, and c3 and d3 are co-directional, where the distribution of the four slip systems before processing is shown in Fig. 3c. The shear strain on a1 (*γ*_*a*1_) is close to *γ*_*a*2_, since non-TD-rotation is negligible. It is the same for c3 and d3. Therefore, only a1 from the a1-a2 set and c3 from the c3-d3 set are used as representatives in the following text. The imbalance of shear strain on slip systems (simply called shear strain imbalance), i.e., difference between cumulative *γ*_*a*1_ and *γ*_*c*3_ (=*γ*_*a*1_ − *γ*_*c*3_), after 1- and 2-cycle is plotted in Fig. 3d. From *t*/*t*0 = 0 to 0.72, *γ*_*c*3_ is higher than *γ*_*a*1_, while *γ*_*a*1_ is dominant from *t*/*t*0 = 0.72 to 1. The alternation between the primary and secondary slip systems divides the M2 band into M2a and M2b (Fig. 3d). The distribution of TD-rotation, shear strain *γ*_*XY*_, and shear strain imbalance (between *γ*_*a*1_ and *γ*_*c*3_) in 2-cycle is similar to that in 1-cycle, since it was conventional rolling (not ARB) in 2-cycle.

**Figure 4:**
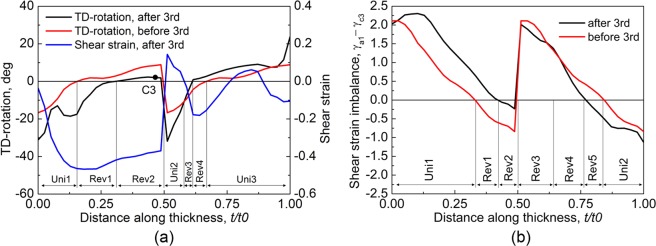
(**a**) Through-thickness shear strain and cumulative TD-rotation, and (**b**) shear strain imbalance between *γ*_*a*1_ and *γ*_*c*3_ in 3-cycle.

ARB was applied from the third cycle. Figure 4a shows the through-thickness cumulative TD-rotation before and after 3-cycle. The distribution of matrix bands before 3-cycle, M1-M2-M1-M2 (not marked in Fig. 4a), clearly reflects the cutting-stacking pattern, i.e., two 2-cycle deformed sheets stacked. The TD-rotation, compared to that before 3-ARB, increased unidirectionally after 3-cycle in some regions marked as ‘Uni’, while the direction of TD-rotation reversed in the other regions (marked as ‘Rev’). The TD-rotation (cumulated in 1- and 2-cycle) dropped in Rev2 and Rev3, implying the crystal orientation rotated toward the initial orientation in 3-cycle, but it is positive both before and after 3-cycle. However, the TD-rotation continued to rotate after passing (1 1 2)[1 1 $\bar{1}$] in Rev1 and Rev4, so the sign of TD-rotation before and after 3-cycle is opposite. The TD-rotation reversed in the Rev2 that had been located at the lower surface of 2-cycle and was moved to the centre of 3-cycle. In contrast, it is unidirectional in Uni2 after the upper surface of 2-cycle being moved to the centre, and the increase of TD-rotation in Uni2 is as high as that at the upper surface of 2-cycle. Additionally, TD-rotation reversal also occurred at non-central thickness positions (e.g., in Rev1). According to the distribution of TD-rotation, the area fraction of positive and negative TD-rotation after 3-cycle is about 50%. The shear strain developed in 3-cycle alone (Fig. 4a) is quite different from that in 2-cycle (Fig. 3b) though the rolling conditions (surface friction and rolling bite geometry) were the same. The difference in shear strain suggests that the previously developed texture affected the following deformation. Figure 4b shows the shear strain imbalance (between *γ*_*a*1_ and *γ*_*c*3_) before and after 3-cycle, which clearly shows the feature of cutting-stacking process, and this feature was preserved after this cycle though the slip system activation changed in some regions marked as ‘Rev’. In Rev1 and Rev5, the shear strain imbalance completely reversed, while it just decreased in Rev2, Rev3 and Rev4, like TD-rotation in Fig. 4a. The distribution of Uni and Rev in Fig. 4a,b does not coincide, but it is Uni near the surface in both of them.

**Figure 5:**
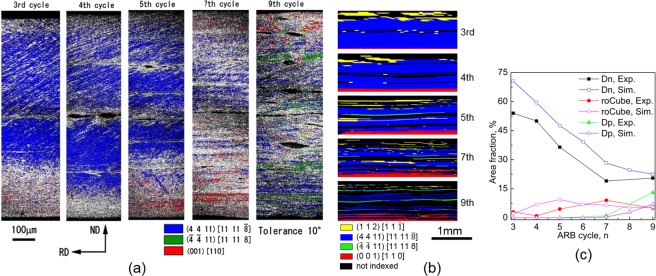
Distribution of texture components through the whole thickness after 3-, 4-, 5-, 7- and 9-cycle in the (**a**) experiment^16^, (**b**) simulation^40^. (**c**) Changes in area fractions of Dn, roCube and Dp^16,40^, where all elements in steady state deformation region were considered. A tolerance of 10° was used to classify crystal orientations in (**a–c**).

The TD-rotation caused texture transition between Cu, Dn, roCube and Dp (Fig. 2). Figure 5a and b show the distribution of texture components through the whole thickness in the experiment^16^ and simulation after various ARB cycles, respectively. The difference in orientation, or misorientaiton, between Cu and Dn is only 8°, which is smaller than the tolerance of 10°. The classified Cu in Fig. 5b is that belongs to (1 1 2)[1 1 $\bar{1}$], but not to (4 4 11)[11 11 $\bar{8}$]. Figure 5c shows the quantitative changes in area fraction of Dn, roCube and Dp. After 3-cycle, the texture component Dn distributed almost through the whole thickness except the upper surface (Fig. 5b), and it replaced Cu and became the major texture component, and the area fraction of Dn reached 70.5% (Fig. 5c). The residual Cu mainly concentrated at the upper surface due to the large negative TD-rotation (Fig. 4a), and the large negative TD-rotation also resulted in unidentified textures near the surface and centre. By contrast, the large positive TD-rotation at the lower surface (Fig. 4a) induced the emergence of roCube (0 0 1)[1 1 0] (Fig. 5b) though this texture component is in an extremely low fraction (2.2%). The Cu component at the upper surface is separated by unidentified texture components, which shows the inhomogeneous texture and deformation at the upper surface of 3-cycle. In contrast, the roCube component at the lower surface and Dn at the sheet inner are hardly divided by other components. The distribution of textures along the RD in Fig. 5b is coarser than that in the experiment (Fig. 5a), since the element size in the simulation is much larger than the formed substructure in the experiment.

**Figure 6:**
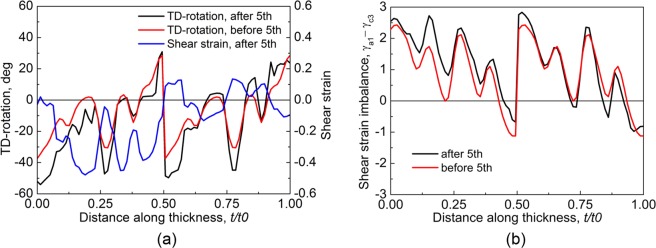
(**a**) Through-thickness shear strain and cumulative TD-rotation, and (**b**) shear strain imbalance between *γ*_*a*1_ and *γ*_*c*3_ in 5-cycle.

After 5-cycle, the TD-rotation and shear strain alternate through the thickness due to the repeated cutting-stacking, as shown in Fig. 6a. Both unidirectional and reversed TD-rotation (Fig. 6a) and shear strain imbalance (Fig. 6b) developed, which is similar to those in 3-cycle (Fig. 4) and 4-cycle (not shown here). The TD-rotation reversed at the upper side of the bonded interface (*t*/*t*0 = 0.5), while its direction is unidirectional at the other side, which has also been observed in 3-cycle (Fig. 4a). The continuous increase of both negative and positive TD-rotation caused the orientations to rotate away from Dn, so the area fraction of Dn decreased to 47.6% after 5-cycle from 70.5% after 3-cycle (Fig. 5c). The Dn was separated by layers of unrecognized texture components (Fig. 5b), where these layers are bonded interfaces. The area fraction of roCube increased slightly to 9.4% (Fig. 5c) due to the positive TD-rotation at the lower surface of 4- and 5-cycle (Fig. 6a). The roCube component was stable at the lower surface (Fig. 5b), but the stability was destroyed after the lower surface (of 4-cycle) being moved to the centre (of 5-cycle), which agrees well with the experimental observation (Fig. 5a). The destruction of roCube at the centre was caused by the reversed TD-rotation (Fig. 6a). The area fraction of negative TD-rotation increased gradually, and became higher than that of positive TD-rotation after 5-cycle. The shear strain imbalance is positive in most regions (Fig. 6b). The through-thickness alternation between Uni and Rev in TD-rotation (Fig. 6a) and shear strain imbalance (Fig. 6b) increased in number because of the repeated cutting-stacking.

**Figure 7:**
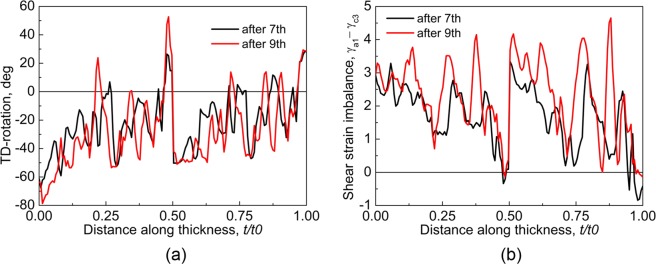
Through-thickness cumulative TD-rotation, and (**b**) shear strain imbalance between *γ*_*a*1_ and *γ*_*c*3_ after 7- and 9-cycle.

After 7- and 9-cycle, the TD-rotation varies sharply in a short distance due to the reduced layer thickness (Fig. 7a). Compared to that after 5-cycle, the TD-rotation increased continuously in the negative direction near the upper surface, and *t*/*t*0 = 0.25 (quarter) and 0.5 (centre), where negative TD-rotation means the crystal orientation rotated toward roCube and Dp (Fig. 2). The texture component Dp started to appear in 7-cycle, but its area fraction is extremely low (Fig. 5c). After 9-cycle, the Dp distributed in thin layers at the centre and quarter (Fig. 5b), and it increased obviously in area fraction, which is similar to that in the experiment (Fig. 5a). The TD-rotation remained almost unchanged in a thin layer at the lower surface, so the area fraction of roCube stayed stable (Fig. 5c). The roCube at the lower surface of 9-cycle is in a layered structure (Fig. 5b), which is probably caused by the sharp change of TD-rotation at bonded interfaces that cumulated in number in the previous cycles. The area fraction of roCube is very low even after 9-cycle, since the ARB was carried out under lubricated conditions. In contrast, the shear texture runs from the surface to even quarter region after unlubricated ARB^30,31^. The texture after 7- and 9-cycle is similar to that after 5-cycle though the main texture component Dn was separated by other texture components, and the area fraction of Dn almost kept unchanged (Fig. 5c). The major texture component is Dn in this study and the corresponding experiment^16^, but it is Cu in ref.^30^. However, both Cu and Dn are considered as rolling texture, and the orientation difference between them is only 8°. Figure 7b shows the shear strain imbalance between *γ*_*a*1_ and *γ*_*c*3_ after 7- and 9-cycle. The slip system a1 is dominated over c3 almost through the whole thickness, which agrees with the experimental observation that slip trace is along a1 direction (Fig. 5a). This agreement is due to that the formation of slip trace is mainly associated with the primarily activated slip systems^32^.

## Discussion

### Comments on CPFEM and ARB FEM model

During processing, the plastic deformation and texture evolution are fully coupled. The starting texture influences plastic deformation, which can be manifested by the different shear strain after each ARB cycle (Figs 3b, 4a and 6a) though the rolling conditions were the same. Meanwhile, the imposed deformation also affects texture evolution, which can be seen from the inhomogeneous through-thickness deformation in 1-cycle (Fig. 3), where the starting texture in 1-cycle is uniform. The coupling between plastic deformation and texture evolution is realized in the CPFEM model, which enabled the prediction of local heterogeneities^26^. In this study, the deformation in a cycle was determined by the currently imposed loadings (reapplied boundary conditions) and previously evolved textures (transferred to this cycle by mapping solution), and accordingly, the plastic deformation and texture evolution were continuously coupled for all ARB cycles. In contrast, the plastic deformation and texture are not fully coupled in other CP models used for ARB simulations (e.g., Taylor model^5^, ALAMEL model^4,6^, and VPSC model^7–12^) due to the homogenization assumption. In this ARB FEM model, the mesh distortion did not cumulate with ARB cycles, since the mesh distortion evolved in a cycle was eliminated by the mapping solution at the beginning of the next cycle, so the convergence problems caused by the mesh distortion was avoided. Through-thickness texture at the meso-scale is the main objective of the current study, so the realistic topology and microstructure of interfaces were not explicitly modelled, but misorientation angles and slip system activation were used to characterize and represent the difference in deformation at the two sides of interfaces. This simplification is similar to that extensively used and widely accepted in polycrystal modelling^18,33,34^, where different initial orientations are assigned to neighbouring grains, but not considering the realistic microstructure of grain boundaries. The good agreement between the simulated textures and experimental observations (Fig. 5) demonstrates that the combination of the CPFEM model and ARB FEM model has captured the main ARB deformation and thus predicted accurate through-thickness texture.

Microstructure refinement can be seen from both experiment (Fig. 5a) and simulation (Fig. 5b) though the EBSD resolution is much higher than the mesh resolution of the simulation. The microstructure has been greatly refined in the experiment, while it is relatively coarse in the simulation, since the substructure is not explicitly modelled in the CPFEM model, where grain refinement is described by misorientation between elements. This difference between experiment and simulation is probably the reason for the area fraction of Dn in the simulation being relatively higher than that in the experiment (Fig. 5c), and this phenomenon (relatively slow microstructure refinement) has been widely observed in CPFEM simulations^26,28,34^.

### Deformation history of a tracer point

**Figure 8:**
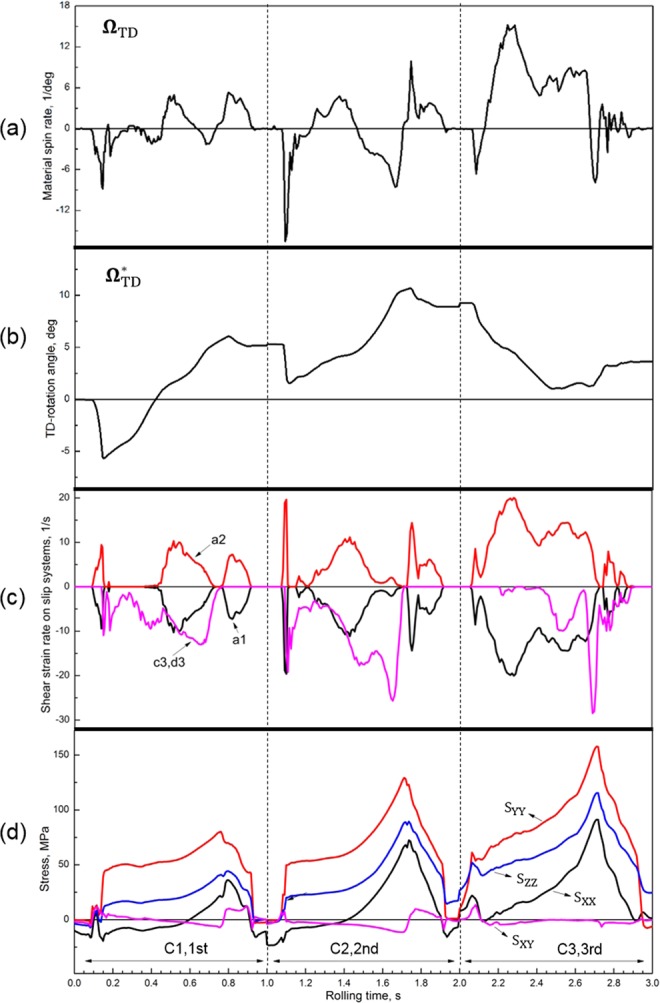
(**a**) Material spin rate, (**b**) lattice spin rate, (**c**) shear strain rate on slip systems, and (**d**) stress at C1, C2 and C3 as a function of rolling time.

The deformation history of a point was traced. This point is C1 and C2 (Fig. 3b) locating near the lower surface in 1- and 2-cycle, respectively, and C2 was moved to C3 (centre) in 3-cycle (Fig. 4a). The deformation of these three tracer elements is shown in Fig. 8 and also summarized in Table 1. According to Eq. (5) in Appendix A, crystal slip on a1-a2 would cause plastic spin **Ω**^p^ about TD (${{\boldsymbol{\Omega }}}_{{\rm{TD}}}^{{\rm{P}}}$) in the positive (‘+’) direction (Fig. 2), while c3-c3 would result in negative (‘−’) ${{\boldsymbol{\Omega }}}_{{\rm{TD}}}^{{\rm{P}}}$, where the distribution of a1*-*a2 and c3-d3 is shown in Fig. 3c. The crystal rotation about TD is represented by lattice spin ${{\boldsymbol{\Omega }}}_{{\rm{TD}}}^{\ast }$ (Eq. (4) in Appendix A), while the rotation of FEM mesh at the macroscopic scale, i.e., *γ*_*XY*_, is expressed by material spin **Ω**_TD_^4^. The relation between them, **Ω**_TD_ = ${{\boldsymbol{\Omega }}}_{{\rm{TD}}}^{\ast }$ + ${{\boldsymbol{\Omega }}}_{{\rm{TD}}}^{{\rm{P}}}$, is expressed by Eq. (4) in Appendix A.

**Table 1:** Deformation at five points, where the shear strain on slip systems, macroscopic shear strain *γ*_*XY*_, and TD-rotation change are the values developed in a single cycle, not cumulated with ARB cycles.

Point	Cycle	Thickness position	Matrix band	*γ*_*a*1_,_*a*2_	*γ*_*c*3_,_*d*3_	${{\boldsymbol{\Omega }}}_{{\bf{TD}}}^{{\bf{P}}}$	Shear strain *γ*_*XY*_ (Ω_TD_)	TD-rotation change (${{\boldsymbol{\Omega }}}_{{\bf{TD}}}^{{\bf{P}}}$)	Relation
						Direction			${{\boldsymbol{\Omega }}}_{{\bf{TD}}}={{\boldsymbol{\Omega }}}_{{\bf{TD}}}^{{\bf{P}}}+{{\boldsymbol{\Omega }}}_{{\bf{TD}}}^{\ast }$
A1	1st	Upper surface	M1	1.2	0.28	+	−0.47	−2.7°	(−) = (+) + (−), $|{{\boldsymbol{\Omega }}}_{{\rm{TD}}}^{{\rm{P}}}|$ < $|{{\boldsymbol{\Omega }}}_{{\rm{TD}}}^{\ast }|$
B1	1st	Centre	M2a	0.73	0.54	+	−0.35	1.5°	(−) = (−) + (+), $|{{\boldsymbol{\Omega }}}_{{\rm{TD}}}^{{\rm{P}}}|$ > $|{{\boldsymbol{\Omega }}}_{{\rm{TD}}}^{\ast }|$
C1	1st	Lower surface	M2b	0.48	0.79	−	−0.038	+5.2°	(−) = (−) + (+), $|{{\boldsymbol{\Omega }}}_{{\rm{TD}}}^{{\rm{P}}}|$ > $|{{\boldsymbol{\Omega }}}_{{\rm{TD}}}^{\ast }|$
C2	2nd	Lower surface	M2b	0.44	0.88	−	0.048	+3.7°	(+) = (−) + (+), $|{{\boldsymbol{\Omega }}}_{{\rm{TD}}}^{{\rm{P}}}|$ < $|{{\boldsymbol{\Omega }}}_{{\rm{TD}}}^{\ast }|$
C3	3rd	Centre	No	0.9	0.4	+	−0.33	−5.3°	(−) = (+) + (−), $|{{\boldsymbol{\Omega }}}_{{\rm{TD}}}^{{\rm{P}}}|$ < $|{{\boldsymbol{\Omega }}}_{{\rm{TD}}}^{\ast }|$

At C1, the activation of a1-a2 caused a positive plastic spin (${{\boldsymbol{\Omega }}}_{{\rm{TD}}}^{{\rm{P}}}$) from 0 s to 0.15 s (Fig. 8c). To meet the large negative material spin (**Ω**_TD_) in Fig. 8a, a large negative lattice spin (${{\boldsymbol{\Omega }}}_{{\rm{TD}}}^{\ast }$) (Fig. 8b) was required according to Eq. (4) in Appendix A. In the next period, from 0.15 s to 0.45 s, the activated slip systems changed to c3-d3, which produced a negative ${{\boldsymbol{\Omega }}}_{{\rm{TD}}}^{{\rm{P}}}$. A positive lattice spin ${{\boldsymbol{\Omega }}}_{{\rm{TD}}}^{\ast }$ evolved to meet the low material spin (**Ω**_TD_). The direction of shear stress *S*_*XY*_ and normal stress *S*_*XX*_ altered in the succeeding period (Fig. 8d), from 0.45 s to 0.75 s, which caused the activation of all four slip systems and produced negative ${{\boldsymbol{\Omega }}}_{{\rm{TD}}}^{{\rm{P}}}$, since the shear strain rate on c3-d3 was higher than that on a1-a2. The **Ω**_TD_ in this period is positive with a low magnitude, so a positive ${{\boldsymbol{\Omega }}}_{{\rm{TD}}}^{\ast }$ was required. In the last period from 0.85 s to 0.92 s, only a1-a2 were activated, since the change of *S*_*XY*_ influenced the resolves shear stress on a1-a2. A positive ${{\boldsymbol{\Omega }}}_{{\rm{TD}}}^{{\rm{P}}}$ developed due to the activation of a1-a2, which was enough to meet **Ω**_TD_ and consequently, ${{\boldsymbol{\Omega }}}_{{\rm{TD}}}^{\ast }$ changed slightly. At C1 in the whole 1-cycle (Table 1), the cumulative shear strain on a1-a2 (*γ*_*a*1,*a*2_ = 0.48) was obviously lower than that on the other set (*γ*_*c*3*,d*3_ = 0.79), so the total plastic spin (${{\boldsymbol{\Omega }}}_{{\rm{TD}}}^{{\rm{P}}}$) after mutual counteraction (between a1-a2 set and c3-c3 set) was negative. A positive lattice spin about TD (5.2°) developed to meet the low negative material spin (*γ*_*XY*_ = −0.038). In M2b (Fig. 3d), in which C1 is located, the plastic spin is slightly larger than lattice spin ($|{{\boldsymbol{\Omega }}}_{{\rm{TD}}}^{{\rm{P}}}|$ > $|{{\boldsymbol{\Omega }}}_{{\rm{TD}}}^{\ast }|$), as shown in Table 1, since the redundant material spin after counteraction between ${{\boldsymbol{\Omega }}}_{{\rm{TD}}}^{{\rm{P}}}$ and ${{\boldsymbol{\Omega }}}_{{\rm{TD}}}^{\ast }$, i.e., *γ*_*XY*_(=−0.038), is negatively low.

The deformation at C2 is similar to that at C1 (Fig. 8 and Table 1), since both C1 and C2 are located at the lower surface. The shear strain imbalance between the two sets of slip systems at C2 is −0.44 (=*γ*_*a*1_,_*a*2−_*γ*_*c*3_,_*d*3_), slightly larger than that at C1 (=−0.31), which is supposed to cause higher plastic spin at C2, but this is actually not the case, since the plastic spin is not only determined by the shear strain imbalance, but also crystal orientation (Eq. (5) in Appendix A). Due to the positive TD-rotation previously developed at C1, the two sets of slip systems became more symmetrical about the TD-ND plane (Fig. 3c), so the counteraction between them became stronger. This is why at C2 plastic spin is smaller than lattice spin ($|{{\boldsymbol{\Omega }}}_{{\rm{TD}}}^{{\rm{P}}}|$ < $|{{\boldsymbol{\Omega }}}_{{\rm{TD}}}^{\ast }|$ in Table 1) and accordingly, *γ*_*XY*_ is positive, although the TD-rotation at C2 (3.7°) is lower than at C1 (5.2°) and imbalance between *γ*_*a*1_,_*a*2_ and *γ*_*c*3_,_*d*3_ at C2 is higher than at C1.

The shear stress *S*_*XY*_ is very low at C3 (Fig. 8), and a1-a2 were highly activated over c3-d3 during the whole rolling period (2.0 s to 3.0 s), as shown in Table 1. A large positive plastic spin (${{\boldsymbol{\Omega }}}_{{\rm{TD}}}^{{\rm{P}}}$) was produced, so a negative ${{\boldsymbol{\Omega }}}_{{\rm{TD}}}^{\ast }$ was required to meet the positive **Ω**_TD_. This indicates that the crystal orientation rotated toward the initial orientation, i.e., crystal rotation reversal.

### Coupled effect of plastic deformation and texture

The deformation at another two points, A1 in M1 and B1 in M2a, in 1-cycle is also listed in Table 1. At A1 (at the upper surface), the shear strain on a1-a2 is greatly larger than *γ*_*c*3_,_*d*3_, which is quite different from that at C1 (at the lower surface), although both A1 and C1 (in 1-cycle) have the same initial orientation. The difference in slip system activation was caused by the different stress, since stress is the only reason for slip system activation (Eq. (6) in Appendix B), while the difference in crystal rotation is also associated with the imposed strain. This is to say that the through-thickness deformation is not uniform, and different loadings (e.g., stress and strain) leads to the inhomogeneous texture evolution. The rotation reversal and alternation of slip system activation at C3 were dependent on its thickness location, where C2 at the lower surface was moved to C3 (at the centre). Rotation reversal has not been found in conventional rolling (2-cycle) that does not involve (cutting-stacking and) thickness position change, while reversed rotation has been observed when ARB was applied in all 3- to 9-cycle. A comparison of the deformation between B1 (1-cycle) and C3 (3-cycle), both at the centre, shows that the TD-rotation is quite different, although both the slip system activation and shear strain at these two points are similar. The difference in TD-rotation was caused by the different starting orientation of B1 and C3, since the starting orientation at C3 is not the initial orientation Cu (due to the previously developed crystal rotation at C1 and C2). This also indicates the importance of coupling between plastic deformation and texture evolution, and the continuity of this coupling is realized by transferring the previously developed textures to the new position (Fig. 1).

**Figure 9:**
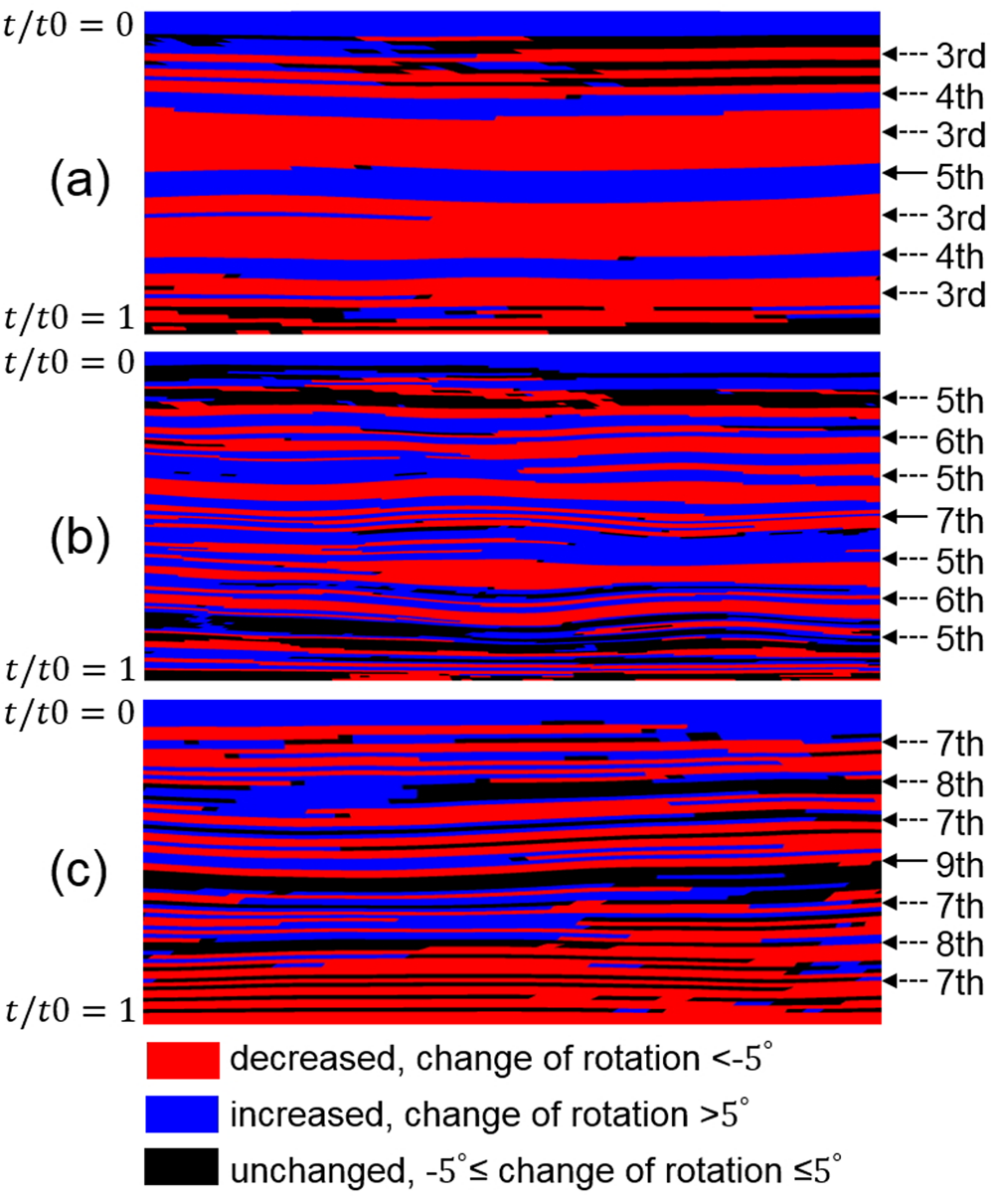
Distribution of crystal rotation change in (**a**) 5-, (**b**) 7-, and (**c**) 9-cycle, where the arrows show the formed bonded-interface in the corresponding cycles.

The texture transition between shear texture and rolling texture has been widely reported in ARB experiments^1,2,4^, and it is claimed^1,31^ that the change between shear deformation (at the surface) and compression (at the centre), or simply called thickness position change here, is the reason for the texture transition. The surface being moved to the centre results in a large change in thickness position, so the transition from shear texture (at the surface of last cycle) to rolling texture (at the centre) is obvious and thus has been widely observed^2,4^. In contrast, the thickness position change at the surface is extremely low, so the shear texture, e.g., roCube in this study, is stable at the surface (Fig. 5b). Due to the small change, texture transition at other thickness positions is not obvious, e.g., the gradual disruption of Dn and formation of roCube in Fig. 5b. The texture transition is associated with crystal rotation. To further reveal crystal rotation transition, the change of crystal rotation in 5-, 7- and 9-cycle was investigated and is shown in Fig. 9. Unlike the reported texture transition^2,4^, the crystal rotation reversal spreads through the whole thickness. The alternation between layers with decreased, increased and unchanged rotation becomes sharp with increasing number of cycles and decreasing thickness of each layer. Texture has almost reached stability after 5-cycle (Fig. 5b), and this stability is due to the dynamic balance between the increased and decreased crystal rotation (Fig. 9a). The through-thickness alternation of deformation and texture in ARB is caused by the thickness position change, which has not been observed in the 1- and 2-cycle of conventional rolling (Fig. 3). The region experiences consecutively unidirectional deformation in ARB reduces after each cycle^2^.

### Effect of interfaces

In addition to the thickness position change, another potential reason for texture transition is the bonded interfaces. The upper and lower surfaces, which experience different deformation (e.g., 3-cycle in Fig. 4), are bonded at the centre of the next cycle, and this results in a large deformation discontinuity, e.g., the sharp change in crystal rotation. The misorientation at the interfaces would affect the deformation on both sides of them^9,35^. Another observation to confirm the effect of bonded-interface is that the crystal rotation on the two sides of 3-cycle interface is different though the two sides were both at the centre (Fig. 4a).

**Figure 10:**
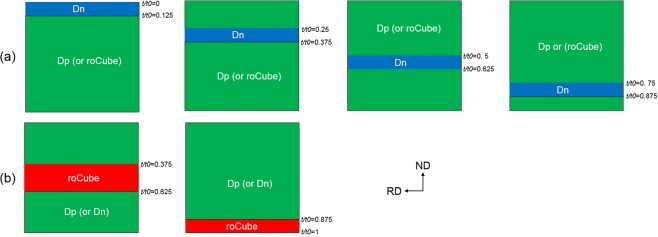
FEM models show the positions of (**a**) Dn, (**b**) roCube, and their neighbourhood.

To study the effect of interfaces, the stability of Dn and roCube, as the main components of rolling and shear texture, respectively, under different interfaces are investigated here during conventional rolling with a 50% reduction. Figure 10 shows the FEM model of bicrystals and interfaces. The Dn layer is located at four positions: *t*/*t*0 = 0 to 0.125, 0.25 to 0.375, 0.5 to 0.625, and 0.75 to 0.875 (Fig. 10a), while the rest of the sheet is Dp or roCube. In Fig. 10b, roCube was placed at two positions: centre (*t*/*t*0 = 0.375 to 0.625) and lower surface (*t*/*t*0 = 0.875 to 1), while the other part is Dp or Dn. The selected orientation (Dn or roCube) was placed at only one position in each simulation case, as shown in Fig. 10. The other simulation conditions are the same as those in the first cycle of this study. This modelling method is similar to that used to study the orientation stability at interfaces of two-phase composites^9,18,35^.

**Figure 11:**
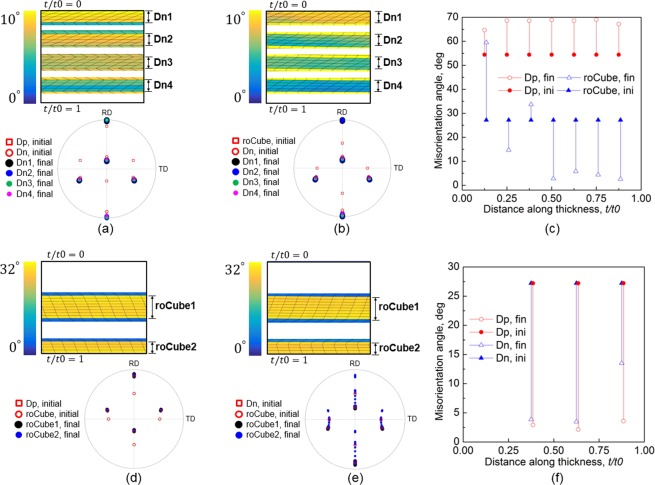
Distribution of deformed FEM mesh and crystal rotation (upper panel), and (1 1 1) pole figures (lower panel) in Dn with neighbouring orientation of (**a**) Dp, (**b**) roCube, and in roCube with neighbouring orientation of (**d**) Dp, (**e**) Dn. Misorientation at interfaces of (**c**) Dn, and (**f**) roCube before and after rolling.

The distribution of crystal rotation and deformed FEM mesh at the four different thickness positions are compared in Fig. 11a,b for Dn, and Fig. 11d,e for roCube. The initial and final crystal orientations are expressed by (1 1 1) pole figures and shown in the lower panel of Fig. 11a,b,d,e. The misorientation angles at all interfaces before and after rolling are shown in Fig. 11c,f. In the case of Dn with neighbourhood of Dp, Dn is very stable at all four positions, as can be seen from the pole figure in Fig. 11a. The shear deformation represented by the deformed FEM mesh at the four positions of Dn is in the same direction (Fig. 11a), but opposite with that in its neighbourhood (Dp), where Dn and Dp are symmetrically distributed about the TD-ND plane. The misorientation at all interfaces increased (Fig. 11c), and the increase near surfaces is slightly lower than that in the inner region. The pole figure in Fig. 11b shows that Dn also remained stable under the effect of neighbouring roCube. However, both decreased and increased misorientation developed (Fig. 11c), and the difference in misorientation change is mainly caused by different crystal rotation in the neighbouring roCube, since the crystal rotation in Dn is very low (the pole figure in Fig. 11b).

In Fig. 11d, roCube rotated as a whole toward its neighbourhood Dp in both the central and lower layers, so the misorientation in Fig. 11f dropped greatly. The shear deformation in roCube and neighbouring Dp is in the same direction (Fig. 11d). When with neighbour of Dn (Fig. 11e), roCube in the central layer also rotated toward Dn. The misorientation at the two interfaces (*t*/*t*0 = 0.375 and 0.625) dropped greatly, which suggests the high stability of the neighbouring Dn. The shear deformation in the central roCube layer is in the same direction as its neighbouring Dn (Fig. 11e), but different from its counterpart in Fig. 11d. As for the roCube at the lower surface (Fig. 11e), obvious texture and deformation gradients developed in it. The deformation and crystal rotation at the bottom region of this layer are in the similar way as those in its counterpart in Fig. 11d, where this layer is close to the surface and greatly influenced by the surface friction. However, the influence of the neighbouring Dn increases as approaches the interface at *t*/*t*0 = 0.875. This roCube layer has to deform in a way to accommodate the deformation in neighbourhood and boundary conditions, and this is why subdivision occurred in this layer. Positive TD-rotation developed near the lower surface (*t*/*t*0 = 1), while negative TD-rotation evolved near the interface (*t*/*t*0 = 0.875). In this case (with neighbouring Dn), the misorientation at *t*/*t*0 = 0.875 is significantly higher than that at the other two interfaces (Fig. 11f).

In Fig. 11, it seems that Dn is not obviously influenced by its neighbourhood, and it is more stable than roCube. The distribution of activated slip systems in Dn, like that in Cu (Fig. 3c), is asymmetrical about the TD-ND plane, and accordingly, one set of slip systems is obviously inclined to the direction of shear stress over the other set. Therefore, a slight change in shear stress does not significantly change the resolved shear stress on the two sets of slip systems and hence does not alter slip system activation, so Dn is not very sensitive to shear stress. However, the four activated slip systems in roCube is symmetrical about the TD-ND plane^36^, so the direction of shear stress is critical. The different deformation between Fig. 11d,e shows the effect of neighbourhood, and it is clear that roCube is not stable even at the lower surface in Fig. 11d,e due to the influence of neighbourhood.

### Discussion on texture and microstructure evolution in ARB

The multi-layer sheet in ARB can be simply analogized by an aggregation of grains in polycrystals. The in-grain deformation of polycrystal is mainly determined by two factors. The first factor is orientations of grains^14^, and the second one is neighbourhood, i.e., grain-interaction. In coarse-grained materials, the deformation in the grain interior is basically determined by the initial orientation (relative to the loading), while grain-interaction is strong near grain boundaries and becomes weak as approaches the inner region, because the grains in coarse-grained materials are large enough to accommodate the deformation gradient. These two factors are crystal orientations and interfaces in the multi-layer sheet of ARB, where the former is strongly influenced by the thickness position change (the crystallographic coordinate system relative to the sample reference system, i.e., external loading), while the interfaces can be simply treated as grain boundaries. Textures rotate into stable positions during deformation, and after being moved to a new position (by mapping solution), the previously developed stable textures become unstable at the new positon that has different external loading. In 3-cycle (Fig. 4a), there is only one interface and accordingly, the *grain size* (layer thickness) is large, so the effect of thickness position change (or crystal orientation relative to loading) is dominant. Therefore, the regions with reversed rotation mainly concentrate near the centre (having large changes in thickness position) though with a small exception (Uni2 in Fig. 4a) at the centre. This exception is probably due to the effect of the interface in 3-cycle, and the division of Uni and Rev is complicated in 5-cycle due to the large number of layers and the increasing effect of interfaces. This phenomenon is more pronounced in the roCube in 9-cycle (Fig. 5b), where it is in a layered structure at the lower surface. The non-roCube between the roCube layers probably reoriented from roCube due to the strong influence of interfaces that had developed in the previously cycles, as observed in ARB deformed two phases materials, like Zr/Nb^37^ and Cu/Nb^9^. This increasing effect of interfaces can be seen from the dominant role of grain-interaction in polycrystals with small grains. As the grain size decreases^34^, the area fraction of grain boundaries increases and inner region decreases, and thus the crystal rotation has to be large to accommodate the orientation gradient over a short distance.

Similar to TD-rotation, both unidirectional and reversed slip system activation have been observed (Figs 4b and 6b), which resulted from the partial change of rolling direction in ARB^2^. The slip system activation is critical to microstructural characteristics and the change of slip system activation would alter microstructure evolution^38^. The influence of rolling direction to slip system activation and microstructure in ARB has not been well researched. However, it has been found in multi-pass unidirectional rolling that the microstructure continued to refine in the way that developed in previous passes^29^, while the previously formed microstructure was destroyed by the next pass in multi-pass reverse rolling. The continuous refinement in the unidirectional rolling and destroyed microstructure in the reverse rolling were due to the same and different activated slip systems, respectively^29^. The alternation of slip system activation could be a potential reason for that ARB is faster than conventional rolling in microstructure refinement^39^.

## Conclusions

1. A CPFEM model was used to study the deformation behaviour in an aluminium single crystal. The CPFEM simulation followed the real multi-cycle ARB process up to 9 cycles.
2. The predicted through-thickness texture has been validated by the experimental observations. A heterogeneous through-thickness deformation was revealed, and alternation of deformation between ARB cycles was also observed. The dynamic balance between the increased and decreased crystal rotation resulted in the stable distribution of textures.
3. The textures developed in previous cycles became unstable after being moved to a new thickness position in ARB, which is manifested by partial rotation reversal and alteration of slip system activation. By contrast, the deformation in conventional rolling is unidirectional due to no change of thickness position.
4. The inhomogeneous through-thickness deformation and cutting-stacking process resulted in large misorientation at the interfaces. The interfaces, like grain boundaries, affected the deformation on the two sides of them, which was investigated by studying the stability of Dn and roCube with different neighbourhood.
5. With increasing ARB cycles, texture evolution in aluminium becomes less sensitive to thickness position change, but it is affected more by interfaces.

## Supplementary information

Appendix: CPFEM theory and hardening model
