# Supplementary material for: A crystal plasticity FEM study of through-thickness deformation and texture in a {112} <111> aluminium single crystal during accumulative roll-bonding
Source: Sci Rep. 2019 Mar 4;9:3401. doi: 10.1038/s41598-019-39039-y (PMC6399260; doi:10.1038/s41598-019-39039-y)
Supplement: Supplementary file 1 — Appendix: CPFEM theory and hardening model [file 41598_2019_39039_MOESM1_ESM.docx]

A crystal plasticity FEM study of through-thickness deformation and texture in a {112}<111> aluminium single crystal during accumulative roll-bonding

Hui Wang^1^, Cheng Lu^1,*^, Kiet Tieu^1^, Guanyu Deng^1^, Peitang Wei^2^, Yu Liu^1^

^1^School of Mechanical, Materials and Mechatronic Engineering, University of Wollongong, New South Wales 2522, Australia

^2^State Key Laboratory of Mechanical Transmission, Chongqing University, Chongqing 400044, China

* hw737@uowmail.edu.au (H.Wang), chenglu@uow.edu.au (C.Lu)

# Appendix: CPFEM theory and hardening model

## Appendix A: Kinematics

The crystal plasticity model in the current research follows the well-recognized kinematical scheme developed by Asaro [^1^](#_ENREF_1) and Peirce [^2^](#_ENREF_2)^,^[^3^](#_ENREF_3). In this scheme, the deformation gradient $\mathbf{F}$ is decomposed into two components as

$\mathbf{F=}\mathbf{F}^{\mathbf{*}}\boldsymbol{\cdot}\mathbf{F}^{P}$ (1)

where $\mathbf{F}^{\mathbf{*}}$ embodies the elastic deformation and rigid body rotation, and $\mathbf{F}^{P}$ consists of crystallographic slip on slip systems. The velocity gradient $\mathbf{L}$ is evaluated from the deformation gradient by

$\mathbf{L}=\dot{\mathbf{F}}\mathbf{F}^{-1}=\mathbf{L}^{*}+\mathbf{L}^{P}$ (2)

The velocity gradient can be uniquely decomposed into a symmetrical part and a skewed-symmetrical part as

$\mathbf{L}=\mathbf{D}+\boldsymbol{\Omega}$ (3a)

$\mathbf{D}=\frac{1}{2}\left( \mathbf{L}+\mathbf{L}^{T} \right)$ (3b)

$\boldsymbol{\Omega}=\frac{1}{2}\left( \mathbf{L}-\mathbf{L}^{T} \right)$ (3c)

where $\mathbf{D}$ and $\boldsymbol{\Omega}$ are called the stretch rate tensor and spin tensor, respectively. $\boldsymbol{\Omega}$ can be represented by the rigid rotation of a finite region or redundant shear strain, and it can also be decomposed into the elastic stretching and lattice rotation part $\boldsymbol{\Omega}^{*}$ and plastic part $\boldsymbol{\Omega}^{P}$, namely

$\boldsymbol{\Omega}=\boldsymbol{\Omega}^{*}+\boldsymbol{\Omega}^{P}$ (4)

$\boldsymbol{\Omega}^{*}$ is due to distortion and rotation of the crystal lattice, which is the reason for texture evolution. The plastic spin $\boldsymbol{\Omega}^{P}$ is caused by the motion of dislocation on slip planes and along slip directions, which is calculated according to

$\boldsymbol{\Omega}^{P}=\sum_{\alpha=1}^{12} \frac{1}{2}\left( s^{(\alpha)}{\cdot m}^{(\alpha)}-m^{(\alpha)}{\cdot s}^{(\alpha)} \right)\dot{\gamma}^{(\alpha)}$ (5)

where $s^{(\alpha)}$ and $m^{(\alpha)}$ are the slip direction and slip plane normal, respectively.

## Appendix B: Hardening model

As for the hardening model, the Bassani-Wu hardening model [^4^](#_ENREF_4)^,^[^5^](#_ENREF_5), a rate-dependent hardening model, is regarded as the best one after comparison of five different hardening models [^6^](#_ENREF_6), and could better describe the work-hardening of FCC crystals. In this hardening model, the shear strain rate $\dot{\gamma}^{(\alpha)}$ is decided by the resolved shear stress $\tau^{(\alpha)}$ on slip system $\alpha$, as expressed by Eq. (6), where $\dot{\gamma}_{0}^{\left( \alpha\right)}$ is the reference value of the shear strain rate, $n$ is the rate-sensitive exponent, and${\tau_{c}}^{(\alpha)}$ is the critical resolved shear stress of the slip system $\alpha$. The values of $\dot{\gamma}_{0}^{\left( \alpha\right)}$, $n$ and ${\tau_{c}}^{(\alpha)}$ are listed in Table 2.

$\dot{\gamma}^{(\alpha)}=\dot{\gamma}_{0}^{\left( \alpha\right)}\mathrm{sgn}(\tau^{(\alpha)})\left| \frac{\tau^{(\alpha)}}{{\tau_{c}}^{(\alpha)}} \right|^{n}$ for $\tau^{(\alpha)}\geq{\tau_{c}}^{(\alpha)}$ (6a)

$\dot{\gamma}^{(\alpha)}=0$ for $\tau^{(\alpha)}<{\tau_{c}}^{(\alpha)}$ (6b)

The $\mathrm{sgn}\left( x \right)=\left\{ \begin{aligned} 1 \mathrm{for} x\geq0 \\ -1 \mathrm{for} x<0 \end{aligned} \right.$ (6c)

The ${\tau_{c}}^{(\alpha)}$ represents the strength of activating the slip system $\alpha$, and its increase rate in value, i.e., ${\dot{\tau}_{c}}^{(\alpha)}$, is determined by:

${\dot{\tau}_{c}}^{(\alpha)}=\sum_{\beta=1}^{N} h_{\alpha\beta}\left| \dot{\gamma}^{(\beta)} \right|$ (7)

where $h_{\alpha\beta}$ is the hardening modulus. As expressed in Eq. (8), the activation of all slip systems would affect the hardening of each slip system. It is self-hardening, i.e., $h_{\alpha\alpha}$, when $\alpha$ is equal to $\beta$, while it is latent hardening $h_{\alpha\beta}$ when $\alpha$ is not equal to $\beta$. The $h_{\alpha\alpha}$ and $h_{\alpha\beta}$ are expressed by:

$h_{\alpha\alpha}=\left[ \left( h_{0}-h_{s} \right)\mathrm{sech}^{2}\left( \frac{\left( h_{0}-h_{s} \right)\gamma^{(\alpha)}}{\tau_{1}-\tau_{0}} \right)+h_{s} \right]\left[ 1+\sum_{\begin{matrix} \beta=1 \\ \beta\neq\alpha\end{matrix}}^{N} f_{\alpha\beta}tanh(\frac{\gamma^{(\beta)}}{\gamma_{0}}) \right]$,$\alpha=\beta$ (8a)

${h_{\alpha\beta}=qh}_{\alpha\alpha}, \alpha\neq\beta$ (8b)

where $h_{0}$ is the hardening modulus after initial yield, $h_{s}$ is the hardening modulus of easy slip, $\tau_{1}$ is the critical stress when plastic flow begins, $\tau_{0}$ is the initial critical resolved shear stress, $q$ is the ratio between latent hardening modulus and self-hardening modulus, and $f_{\alpha\beta}$ means the interaction between slip system $\alpha$ and $\beta$. The value of $f_{\alpha\beta}$ is determined by the relative position of two slip systems, and thus five constants of $f_{\alpha\beta}$ exist. The parameter $f_{\alpha\beta}$ is chosen as: $a_{1}=a_{2}{=a}_{3}=1.75$, $a_{4}=2$ and $a_{5}=2.25$ according to the study in Ref. [^7^](#_ENREF_7). Other material parameters in Eq. (6) and (8) are listed in Table 2, which were evaluated by fitting the simulated stress–strain curve with the experimental results of an aluminium single crystal under plain strain compression [^8^](#_ENREF_8). The three elastic moduli are $C_{11}=112,000 MPa$, $C_{12}=66,000 MPa$ and $C_{44}=28,000 MPa$.

Table.2. Parameters used in the Bassani-Wu hardening model.

| $n$ | $\dot{\gamma_{0}} (s^{-1})$ | $h_{0}$ (MPa) | $h_{s}$ (MPa) | $\tau_{1}$ (MPa) | $\tau_{0}$ (MPa) | $q$ |
| --- | --- | --- | --- | --- | --- | --- |
| 300 | 0.0001 | 100 | 0.01 | 6.3 | 6 | 1 |

## References

1 Asaro, R. J. CRYSTAL PLASTICITY. *J. Appl. Mech.* **50**, 921-934 (1983).

2 Peirce, D., Asaro, R. J. & Needleman, A. An analysis of nonuniform and localized deformation in ductile single crystals. *Acta Metall.* **30**, 1087-1119 (1982).

3 Peirce, D., Asaro, R. J. & Needleman, A. Material rate dependence and localized deformation in crystalline solids. *Acta Metall.* **31**, 1951-1976 (1983).

4 Wu, T.-Y., L.Bassani, J. & Laird, C. Latent Hardening in Single Crystals I. Theory and Experiments. *Pro. R. Soc. A* **435**, 20 (1991).

5 L.Bassani, J. & Wu, T.-Y. Latent Hardening in Single Crystals II. Analytical Characterization and Predictions. *Pro. R. Soc. A* **435**, 21 (1991).

6 Lin, G. & Havner, K. S. A comparative study of hardening theories in torsion using the Taylor polycrystal model. *Int. J. Plast.* **12**, 695-718 (1996).

7 Franciosi, P., Berveiller, M. & Zaoui, A. Latent hardening in copper and aluminium single crystals. *Acta Metall.* **28**, 273-283 (1980).

8 Liu, Q., Maurice, C., Driver, J. & Hansen, N. Heterogeneous Microstructures and Microtextures in CubeOriented Al crystals after channel die compression. *Metall. Mater. Trans. A* **29**, 2333-2344 (1998).
